# Psychological resilience and cognitive frailty progression among older adults: Evidence from China in 2002-2018

**DOI:** 10.1093/geroni/igaf122.1611

**Published:** 2025-12-31

**Authors:** Ruoxi Ding, Yanan Luo

**Affiliations:** Peking University, Beijing, Beijing, China (People’s Republic); Peking University School of Public Health, Beijing, Beijing, China (People’s Republic)

## Abstract

**Objectives:**

Psychological resilience (PR) may protect against cognitive frailty, yet its relationship remains unexplored. This study examines the association between PR and cognitive frailty progression among older adults in China using 16 years of nationally representative cohort data.

**Methods:**

Data from the Chinese Longitudinal Healthy Longevity Survey (CLHLS, 2002–2018) were analyzed. Cognitive frailty was defined as coexisting physical frailty and cognitive impairment without dementia. Cognitive function was assessed using the Chinese Mini-Mental State Examination (CMMSE), and physical frailty was measured through daily living, instrumental activities, and functional limitations. Fixed-effect models, Latent Class Growth Models, and multinomial logistic and Cox regression analyses were used to explore PR trajectories and their links to cognitive frailty progression.

**Results:**

A 1-point increase in PR score reduced cognitive frailty risk by 9% (RRR=0.91, 95% CI: 0.90, 0.93), with stronger effects in younger, non-illiterate, and married individuals. Compared to the high-level stable PR group, those with moderate-level declining or low-level rising PR faced higher risks of mild cognitive impairment (MCI) with accelerated frailty (RRR=1.34, 95% CI: 1.15, 1.56; RRR=2.10, 95% CI: 1.39, 3.16, respectively) and high-probability MCI and frailty trajectories (RRR=1.59, 95% CI: 1.26, 2.02; RRR=3.49, 95% CI: 2.13, 5.72).

**Conclusions:**

Higher PR levels are linked to reduced cognitive frailty risk, particularly among younger, married, and non-illiterate older adults. Lower PR trajectories predict greater cognitive and physical decline, underscoring the importance of PR interventions in secondary prevention strategies for cognitive impairment and disability, and in promoting healthy aging.

